# The impact of soil erosion on soil-related ecosystem services: development and testing a scenario-based assessment approach

**DOI:** 10.1007/s10661-020-08814-0

**Published:** 2021-05-14

**Authors:** Bastian Steinhoff-Knopp, Tinka K. Kuhn, Benjamin Burkhard

**Affiliations:** 1 Institute of Physical Geography and Landscape Ecology, Leibniz University Hannover, Schneiderberg 50, 30167 Hannover, Germany; 2 Leibniz Centre for Agricultural Landscape Research ZALF, Eberswalder Straße 84, 15374 Müncheberg, Germany

**Keywords:** Water erosion, CICES, Soil natural capital, Landscape, Control of erosion rates, Soil retention

## Abstract

The ecosystem service (ES) approach usually addresses soil erosion as the regulating service *control of erosion rates* or *soil retention.* In addition to the assessment of this regulating ES, mitigated impacts on *soil-related ES* by preventing soil erosion can be assessed. This study presents a scenario-based approach for the assessment of the impact of soil erosion on soil-related ES. The assessment approach was tested in agricultural landscapes in Northern Germany, combining mapping and assessment of *soil-related ES*. In six scenarios, the degradation of soils due to soil erosion was simulated by the calculation of soil profile reductions. The scenarios represent two levels of impact with three time steps (+50, +100, +150 years). In the scenarios for the *structural impact*, the *potential soil erosion rates* were extrapolated into the future to generate spatially explicit information on degraded soils. In the scenarios for the *mitigated impact*, the *actual soil erosion rates* were extrapolated. Four *soil-related ES* were assessed for the initial state and the scenarios crop provision, water filtration, water flow regulation and fresh water provision. The comparison of the potential service supply of the four soil-related ES in the scenarios enabled the assessment of the long-term effect of the ES *control of erosion rates*. The mitigated reduction in the potential service supply for three of the considered ES (crop provision, water filtration, water flow regulation) is large and highlights the importance of sustainable soil management. Contrary to this, the ES fresh water provision benefits of erosion-induced soil profile reductions.

## Introduction

The capacity of agricultural ecosystems to provide ecosystem services (ES) is directly related to the condition of soils and their properties and functions (Dominati et al. 2010; see Box 1 for definitions). Therefore, soil protection is mandatory for sustaining the capacity of agroecosystems to supply provisioning and regulating ES. In addition, soil should be protected to preserve related cultural ES (e.g. soils act as a geoarchive). The structure and condition of soils are relevant factors determining soil functions and the related potential of many ecosystems to provide provisioning and regulating ES (Daily et al. 1997; Adhikari and Hartemink 2016). From a functional point of view, the potential supply of many ES (e.g. food production in terrestrial ecosystems as a provisioning service) depends largely on soil and its properties. These services are often named *soil ES* (e.g. Adhikari and Hartemink 2016; Jónsson et al. 2017) or *ES of soils* (e.g. Dominati et al. 2010). Considering that ES are defined as human benefits from ecosystems, we prefer the term *soil-related ES* (see Box 1 for definition)*.* This emphasises that the respective service is provided by an ecosystem, which is considered a holistic entity of abiotic and biotic components that are interacting with each other. Soils are significantly important and functionally required for soil-related ES supply. Fundamentally important for the mapping and assessment of soil-related ES are spatially explicit data on soil properties (maps/geodata on soil properties and functions, soil profile descriptions).

**Box 1 Definitions**

**Table:** 

**• Soil-related ecosystem service:** Ecosystem service whose supply is directly and quantifiably controlled by soil properties, processes, and functions. **• Soil natural capital:** Stocks of mass, energy, and their organisation (entropy) that forms soils (Robinson et al. 2009). **• Soil properties:** The physical, chemical, and biological characteristics of a soil, which can be described or measured by field or laboratory observations. Differentiated into “inherent” and “dynamic” (Robinson et al. 2009) / “manageable” (Dominati et al. 2010) properties. **• Soil functions:** Subset of the interactions between biophysical structures, biodiversity, and ecosystem processes that underpin the capacity of a soil to support ecosystem service supply (after TEEB 2010). **• Control of erosion rates (CER):** ES controlling or preventing soil loss. Mainly provided by vegetation covering the soil. **• Structural impact:** Effect on environmental resources when no mitigation is provided, resulting from threats and/or ecosystem processes. Example: soil loss when ES *control of erosion rates* is provided (Guerra et al. 2014). **• Mitigated impact:** Effect on environmental resources when mitigation is provided, resulting from threats and/or ecosystem processes. Example: Remaining soil loss when ES *control of erosion rates* is provided (Guerra et al. 2014).	

### Soil natural capital and soil-related ecosystem services

Robinson et al. (2009) define the supply of soil-related ES as the flow of soil natural capital (see Box 1 for definition) formed by the stocks of biotic and abiotic mass interrelated through energy and organisation. These stocks were generated in the process of soil formation, determined by the soil formation factors’ parent material, topography, climate, organisms, and time (Jenny 1941). Soil properties characterise the condition of soil natural capital stocks and therefore the potential to support ES supply. Soil formation and supporting processes enrich the stock while degrading processes, like soil erosion or compaction, can enable the depletion of soil natural capital.

The natural capital stock of soil can be altered by natural and anthropogenic pressures that influence the speed and nature of soil processes (Dominati et al. 2010). Human impacts through land use and applied farming practices can both foster and alter the condition of soils. Explicit threats to soils that lead to soil degradation with negative impacts on the supply of soil-related ES include soil erosion, decline in soil organic matter, soil contamination, soil sealing, soil compaction, decline in soil biodiversity, salinisation, floods, and landslides (European Union 2006a). At a global scale, the preservation of soils is addressed by the UN-FAO global soil partnership (Montanarella 2014) and indirectly within the UN Sustainable Development Goals (UN General Assembly 2015). These initiatives value the contributions of soils to ES supply, for environmental sustainability and human welfare goals (Adhikari and Hartemink 2016).

### Soil erosion as a threat to soils: aspects and concepts

Soil erosion by water is a major threat to soils in Central Europe (Panagos et al. 2015). The Thematic Strategy for Soil Protection of the European Commission lists soil erosion as the most relevant soil degradation process in EU member states and underpins the importance of soil conservation (European Union 2006a). Various frameworks and concepts to model and assess soil erosion and its impact were developed to address this problem. Verheijen et al. (2009, p. 23) reviewed the concept of tolerable soil erosion, defined as “any actual soil erosion rate at which a deterioration or loss of one or more soil function does occur”, and calculated mass balances between soil erosion and soil formation rates. Soil sustainability is affected in cases where soil erosion rates are higher than soil formation rates. A similar framework developed by Robinson et al. (2017) also proposed mass balances to account the state and change in soil natural capital as a base for further assessments.

Concepts for the economic valuation of soils are discussed in detail in Robinson et al. (2014), whereby the authors conclude that the cost valuation of soil erosion is the most common approach. Pimental et al. (1995) assessed the total costs of wind and water erosion in the USA to be US$ 44.4 billion. The European Union (2006a) estimated in an impact assessment the annual costs of soil erosion in the EU member states to be € 0.7 to 14 billion. Utilising modelled soil loss rates, Panagos et al. (2018) estimated the impact of soil erosion by water on crop production in the EU and concluded that the related annual costs of losses in agricultural productivity due to soil erosion add up to € 1.25 billion. These studies show that the inclusion of economic aspects in soil degradation assessments can be a powerful step to emphasise the impact of soil erosion and other forms of soil degradation on human well-being.

In ecosystem service approaches and assessments, soil erosion is mainly addressed by the regulating ES *control of erosion rates* (CER, see Box 1 for definition)*.* CER ES describes the reduction of soil loss by ground cover realised by plants (CICES 5.1; Haines-Young and Potschin 2018). The framework for the assessment of regulating service supply and the CER ES developed by Guerra et al. (2014) defines the potential soil loss as *structural impact* and the actual soil loss as *mitigated impact*. The CER service supply is the difference between the potential and the actual soil loss (resp. the difference between structural and migrated impact) and mainly a function of ground cover by plants and the soil tillage management.

As shown by Guerra et al. (2014, 2016), this framework can be operationalised by modelling the potential and actual soil loss utilising the Universal Soil Loss Equation USLE (Wischmeier and Smith 1978) or one of its many adaptions. Steinhoff-Knopp and Burkhard (2018a) included soil loss rates obtained from long-term monitoring sites. The mitigated soil loss rate in *t*/*(ha ∙ a)* was used as an indicator for the respective ES supply in this study.

### Combining soil erosion and the loss in soil-related ecosystem services in scenarios

Soil erosion leads to soil degradation: Soil material, and by this organic carbon and nutrients, gets lost, resulting in reduced soil natural capital and thereby in a decrease in soil-related ES supply. For the assessment of decreases in soil-related ES due to soil erosion, a scenario-based approach can be helpful: Soil loss rates can be projected into the future to simulate degraded soils with a reduced potential to supply soil-related ES. The scenarios should provide explicit soil profile descriptions and related data on soil properties. Based on such simulated changes, soil-related ES and their reduced supply in the scenarios can be assessed. Comparing scenarios for potential and actual soil loss enables the assessment of long-term effects of CER ES on soils and their services.

The aim of this study is to develop and operationalise such an approach for the assessment of the long-term effects of CER ES on soils and the services they provide. The investigation areas represent three typical landscapes prone to soil erosion by water in Northern Germany. To test the approach, assessments of four soil-related ES in six scenarios representing soil degradation stages in 50, 100, and 150 years are carried out. The scenarios correspond to two levels of impact, extrapolating observed actual and potential soil loss rates into the future. The levels of impact in the scenarios represent the *structural impact* of soil erosion and the *mitigated impact.* The objective is to evaluate the long-term effect of CER ES by comparing the potential supply of ES co-provided by soils in the scenarios.

Based on this aim, the following questions will be answered:

- Is the proposed assessment approach suitable to indicate the long-term effect of the ES CER?
- Are there differences between the potential supply of soil-related ES of the scenarios for the actual and potential soil loss?
- Do the results for four selected soil-related ES and the three investigation areas differ?

## Material and methods

### Approach for the assessment of the mitigated loss in soil-related ecosystem services using the example of the regulating service *control of erosion rates*

**Fig. 1:**
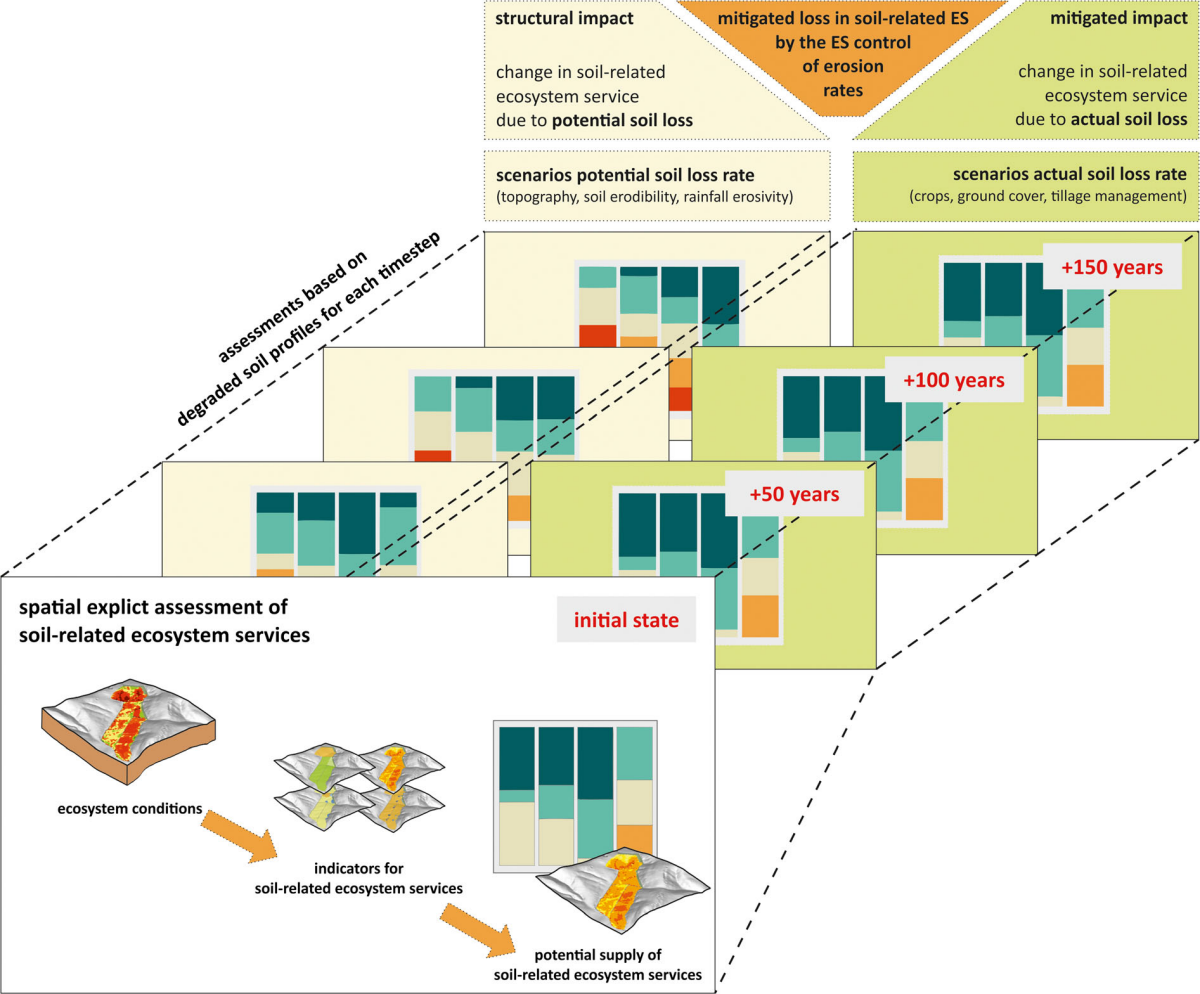
General approach for the assessment of the mitigated loss of soil-related ecosystem services by the regulating ecosystem service “control of erosion rates”

The basic concept of the assessment approach is the spatially explicit comparison of the potential soil-related ES supply in scenarios representing different future states of soil degradation. Figure 1 graphically summarises the approach. Apart from the conditions in the initial state, three future time steps (+50, +100, and +150 years) and two levels of impact were considered, resulting in six explicit scenarios. In the scenarios for the *structural impact*, the *potential soil erosion rates* (SL_pot_) were projected into the future. Scenarios representing the *mitigated impact* assumed that the *actual soil erosion rates* (SL_act_) will be constant. Therefore, scenarios for the *structural impact* represent the hypothetical soil loss that would occur when soils are not protected by vegetation, while the scenarios for the *mitigated impact* assumed that the current agricultural management practices will be continued. Simulating the degradation for each scenario, the initial soil profiles are reduced according to the scenario-specific spatially explicit loss rates. In order to compare the reduction in the potential ES supply in the scenarios with different levels of impact, the potential supply of soil-related ES for each scenario and the initial state must be assessed. Comparing the potential ES supply in the scenarios will finally enable the assessment of the mitigated decrease in soil-related ES by the ES *control of erosion rates (CER).*

In this study, the assessment approach was spatially explicitly operationalised for 465.5 ha of cropland in Northern Germany. The potential soil loss was modelled with the German standard version of the USLE (Deutsches Institut für Normung e.V. 2017). The actual soil loss rates were obtained from the Lower Saxonian soil erosion monitoring programme (Steinhoff-Knopp and Burkhard 2018b). For the assessment of potential ES supply, four soil-related ES were selected: crop provision (CP), water filtration (WF), water flow regulation (WFR), and fresh water provisioning (FWP). The methods for the calculation of the soil loss rates and the soil profile reduction, definitions of the four assessed soil-related ES, and their indicators are described in the next paragraphs.

### Study area and data on actual and potential soil loss

**Fig. 2:**
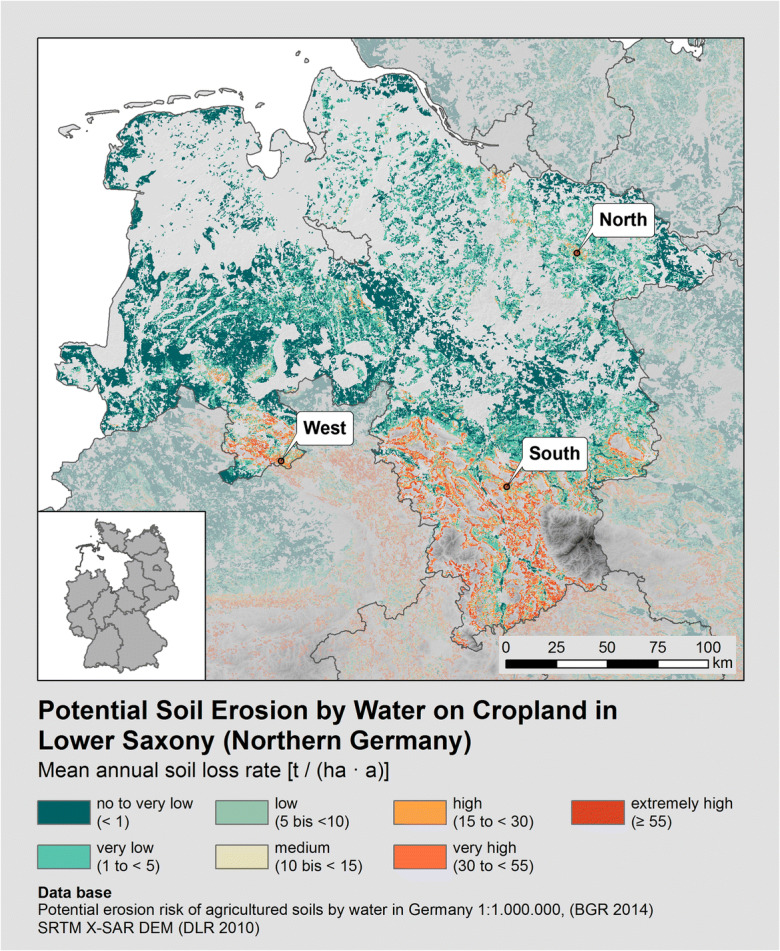
Potential soil erosion by water on cropland in Lower Saxony and investigation areas of the Lower Saxonian soil erosion monitoring (Regions North, West, and South). Map compiled based on the map of the potential erosion risk of agricultural soils by water in Germany at the scale of 1:1.000.0000 (BGR 2014) and SRTM X-SAR DEM (DLR 2010)

The study area has been defined by the Lower Saxonian soil erosion monitoring programme and the monitored cropland within this programme (Steinhoff-Knopp and Burkhard 2018b). 465.5 ha in three regions of Lower Saxony in Northern Germany with medium-to-high water erosion risk have been monitored since the year 2000 (Fig. 2). The cropland under investigation in the three regions (North, West, and South) is typical for landscapes prone to soil erosion in Northern Germany. The study sites are specific in their landscape composition, soil conditions, cultivated crops, farm and tillage management, and their average potential and actual soil loss rates (see Table 1). Due to the origin of the soil substrates (loess and sandy loess), most of the soils in the study area are highly erodible.

**Table 1:** Information on the investigation areas North, West, and South in Northern Germany (Sources: Steinhoff-Knopp and Burkhard 2018a, 2018b)

Region	Area (*ha*)	Fields (*n*)	Mean actual loss rate (SL_act_) (*t*/*(ha* **∙** *a)*)	Mean potential loss rate (SL_pot_) (*t*/(*ha ∙ a*))	Dominant crops
North	137.7	22	1.47	11.20	Winter wheat, winter barley, sugar beet, potato
West	28.4	10	0.73	21.99	Winter wheat, rapeseed, winter barley, maize
South	298.3	54	0.65	20.73	Winter wheat, sugar beet, rapeseed, winter barley

The aims of the long-term monitoring programme are to quantify soil loss on the 86 agricultural fields under observation, determine the frequency of soil erosion events, and create time series. In addition, soil losses under different crops and management systems can be evaluated. The quantification of the soil loss is based on continuous measurements of erosion damages in field surveys, which are combined with farmer surveys on their management practices. The methods are described in detail in Steinhoff-Knopp and Burkhard (2018b).

**Fig. 3:**
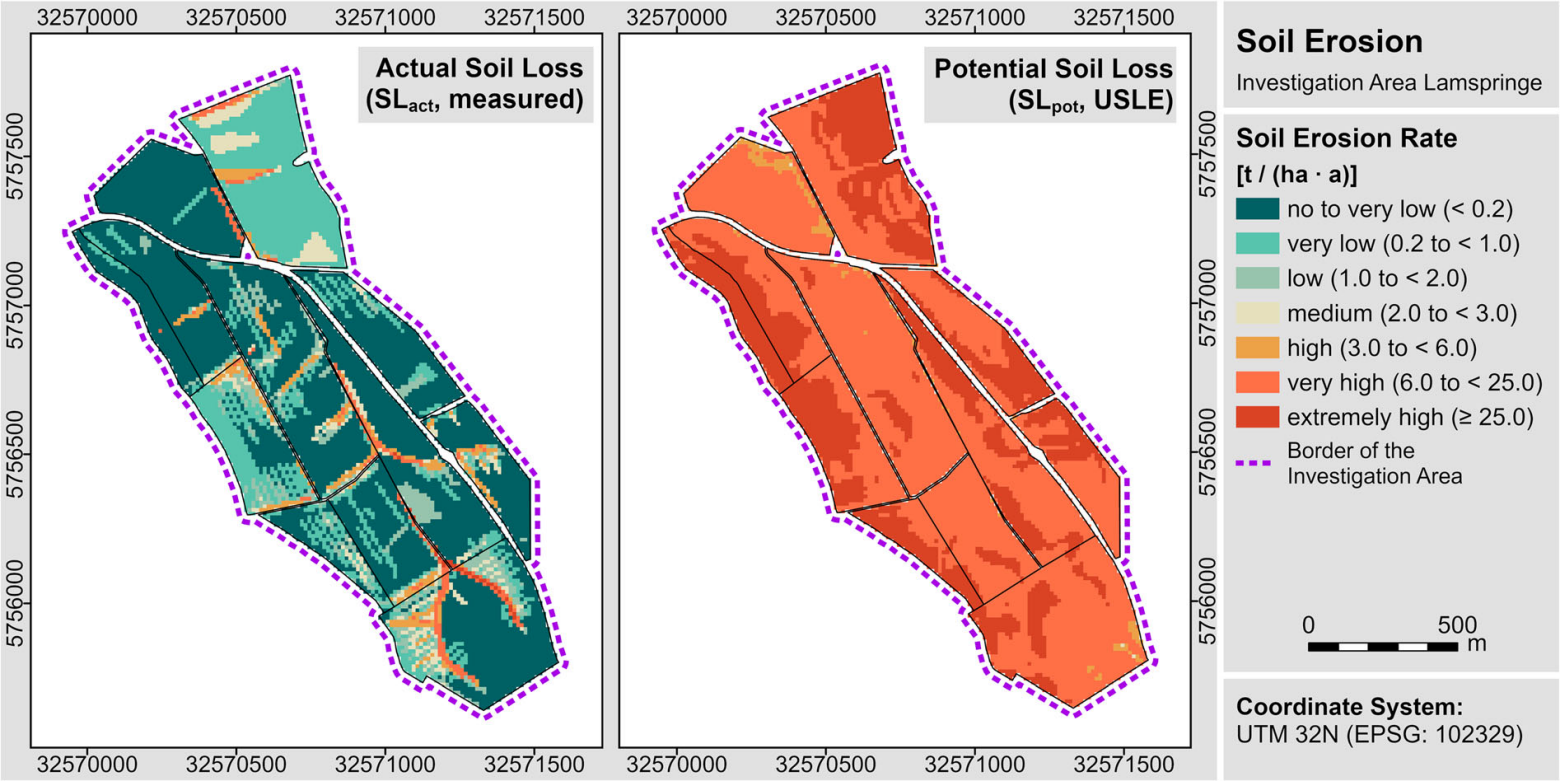
Exemplary maps of the investigation area Lamspringe (Region South), presenting the spatial datasets “actual soil loss” (SL_act_, measured in the *Lower Saxonian soil erosion monitoring programme*) and “potential soil loss” (SL_pot_, modelled with USLE)

The key dataset obtained in the monitoring is the measured *actual soil loss* (SL_act_). Actual soil loss (SL_act_) denotes the mean annual soil loss under the current management conditions in *t/(ha ∙ a)* that was measured in 17 years of monitoring (2000 to 2016). GIS-methods (Geographical Information System-methods) were used to combine the spatially explicit mapped soil erosion features to high-resolution maps of soil loss. For this study, a rasterised version of the spatial data on actual soil loss rates, originally published in Steinhoff-Knopp and Burkhard (2018b), was used. This spatial data was also used in Steinhoff-Knopp and Burkhard (2018a) and has a grid resolution of 12.5 m, resulting in a total of 29,181 explicit raster cells (see Fig. 3). Table 1 provides information on the average actual (SL_act_) and potential loss rates (SL_po__t_) of the three investigation areas. Detailed information on methods and the spatial variability of the loss rates are documented in Steinhoff-Knopp and Burkhard (2018a, 2018b).

For the calculation of the *potential soil los*s (SL_pot_) [*t/(ha* ∙ a)], the German standard of the USLE (DIN 19708 Deutsches Institut für Normung e.V. 2017) was applied. Grids for the 465.5-ha cropland of the study area in a resolution of 12.5 m (29,181 raster cells) were generated (see Fig. 3). The following equation defines the calculation and factors used:

SL_pot_ = *R* ∙ *K* ∙ *S* with:

*R:* rainfall and runoff erosivity factor [*N*/*(ha* ∙ *a)*]

data source: weather stations in the study area, operated by the Landesamt für Bergbau, Energie und Geologie (LBEG), and Leibniz University Hannover (LUH)

*K:* soil erodibility factor [*(t* ∙ *h)/(ha* ∙ *N*)]

data source: Lower Saxonian Soil Map (Scale 1:50,000) (Landesamt für Bergbau, Energie und Geologie (LBEG) 2017)

*S:* slope factor [−]

data source: Digital Elevation Model (DEM) in 12.5 m resolution (Landesbetrieb Landesvermessung und Geobasisinformation (LGLN) 2013)

### Generating data on degraded soils: soil profile reductions

**Fig. 4:**
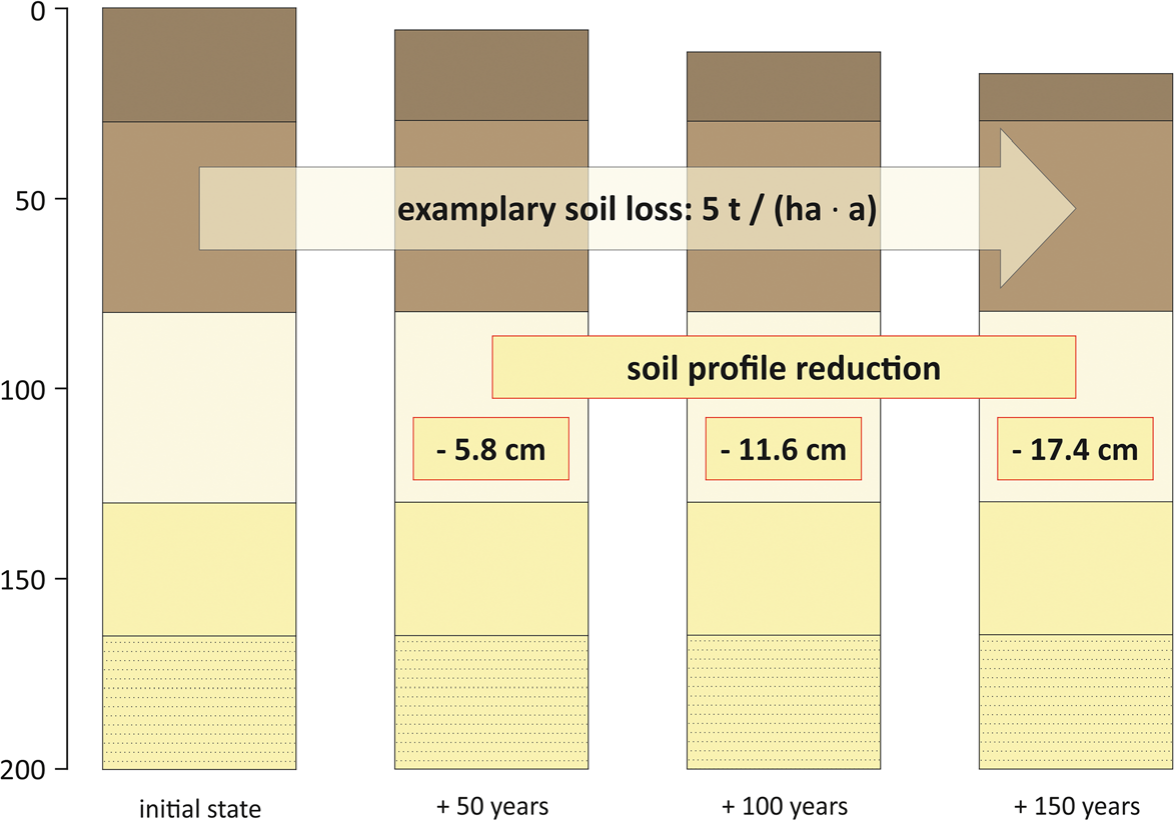
Reduction of an example soil profile for the scenario time steps 0, +50, +100, and +150 years. Assumptions: soil loss of 5 *t/(ha ∙ a)*, bulk density of 1.45 *g/cm*^*3*^. Colours of the horizons in the soil profiles are only illustrative

To create the spatially explicit scenarios for degraded soil conditions, a simple approach was applied: Assuming that no future changes will occur in agricultural management, soil tillage, and climate, the data on actual and potential soil loss rates (SL_act_ and SL_pot_) were projected into the future. The spatially explicit soil losses were calculated for the six scenarios. Resedimentation of eroded soil material within the monitoring areas was not included. Considering a mean soil bulk density of 1.45 *g/cm*^*3*^, the soil losses were recalculated to the reduction of the soil profiles. Figure 4 illustrates the profile reduction at the three scenario time steps (+50, +100, and +150 years) for an exemplary soil loss of 5 *t/(ha ∙ a)*. Combining this data with the specific soil horizon information for the initial state of the soils obtained from the Lower Saxonian soil map (scale 1:50,000) (Landesamt für Bergbau, Energie und Geologie 2017), spatially explicit information on the degraded soils for all scenarios was generated. The results are seven datasets (initial state and six scenarios) in a resolution of 12.5 m (29,181 raster cells per dataset) with detailed soil profile descriptions containing information on soil properties for each soil horizon.

### Soil-related ecosystem services and their indicators

Based on the Common International Classification of Ecosystem Services (CICES 5.1, Haines-Young and Potschin 2018), four soil-related ES with strong linkages to soil properties, conditions and functions were chosen for quantification of the potential ES supply: crop provision (CP), water filtration (WF), water flow regulation (WFR), and fresh water provision (FWP) (Table 2). Each service was quantified for the six scenarios and the initial state by an indicator based on soil and climate data. The methods for the indicator quantification are described in detail in Müller and Waldeck (2011). In this compendium, published by the Lower Saxonian Authority for Mining, Energy and Geology (LBEG), methods for soil conservation utilised as a standard in planning and consulting in Lower Saxony are compiled.

**Table 2:** Quantified soil-related ecosystem services, their indicators, and matching CICES V5.1 classes (Haines-Young and Potschin 2018; Haines-Young and Potschin-Young 2018)

Soil-related ES	Matching CICES V5.1 classes (simplified name) with class code	CICES V5.1 section	Example goods and benefits	Applied indicator	Indicator specification
**Crop provision (CP)**	Cultivated terrestrial plants for nutrition, materials and energy (1.1.1.1; 1.1.1.2; 1.1.1.3)	Provisioning (biotic)	Harvested crop	Potentially available yield	Potential yield of winter barley in *t/(ha ∙ a)*
**Water filtration (WF)**	Bio-remediation of wastes (2.1.1.1); biotic filtration, sequestration and storage of waste (2.1.1.2); abiotic filtering, sequestration and storage of waste (5.1.1.3)	Regulation and maintenance (biotic and abiotic)	Biogeochemical effects of reduced dissolved chemicals (e.g. heavy metals or pesticides) in ground water for drinking	Nitrate leaching vulnerability	Soil water exchange rate in *%/a*
Water flow regulation (WFR)	Hydrological cycle and flood control (2.2.1.3); control of liquid flows (5.2.1.2)	Regulation and maintenance (biotic and abiotic)	Mitigation of damage as a result of reduced in magnitude and frequency of flood/storm events	Water storage capacity of the soil	Amount of potential storable water in the soil in *mm*
**Fresh water provision (FWP)**	Ground (water for drinking (4.2.2.1)	Provisioning (abiotic)	Water for drinking in the local public supply system	Percolation rate	Percolated water per year in *mm/a*

For the assessment of the potential ES supply at the initial state, detailed soil information (spatially explicit descriptions of the soils with information on their properties for each soil horizon) were taken directly from the Lower Saxonian Soil Map (Landesamt für Bergbau, Energie und Geologie (LBEG) 2017). For the six scenarios, the generated data on degraded soil profiles (see previous paragraph) was used. Climate data was obtained from the Climate Data Center of the German Meteorological Service (DWD Climate Data Center 2018a, 2018b).

*Crop provision* (CP) is a provisioning ES that describes the benefits of harvested crops grown in cultivated ecosystems. The most obvious indicator for this ES is crop yield per area per year (Layke et al. 2012). In modern agriculture, farmers manage and highly modify ecosystem functions through inputs of, for instance, fertiliser, pesticides, herbicides, irrigation water, energy, and labour. Hence, the amount of harvested crop is not only a function of ecosystem conditions but is also (as with other co-produced ES) strongly dependent on anthropogenic system inputs (Power 2016; Burkhard et al. 2014; Zhang et al. 2007). Nevertheless, climate, soil, and other ecosystem conditions are still relevant to define the potential to produce crops. For example, the amount of plant available water that can be stored in soils is a fundamental parameter for the potential crop yield. The amount and composition of clay minerals, as well as the alkalinity of soils, define the availability of nutrients, and the mineral composition and content are also relevant for the natural fertility of a site. In addition, the climate defines the duration of the growing season and the timing and amount of precipitation. Using empirical models based on yield data, the potential yield of a specific crop in relation to the climate and soil conditions can be quantified (Palosuo et al. 2011).

In crop provision ES assessments, ecosystem (“natural”) contributions should be separated from human inputs. Accordingly, Bastian et al. (2013) combined soil, climate, and topography data to create a map of the “natural yield potential” as an indicator for the potential crop provision. The “Muencheberger Soil Quality Rating” is a rating system that assesses the long-term soil quality and provides a rough estimation of the potential crop yield (Mueller et al. 2013). The indicator *potential available yield* applied in this study is an empirically derived formula estimating the potential yield of winter barley in *t/(ha ∙ a)*. The most important input parameters are the yearly water balance (budget of precipitation and evapotranspiration), the plant available water storage capacity in the rooted soil, and the amount of clay in the soil (see Müller and Waldeck (2011) for details).

*Water filtration* (WF) is a regulating ES that describes the transformation of biochemical inputs into the ecosystem by soils. The related service *water purification* as proposed by Burkhard et al. (2014) includes the ES water filtration and similar purification effects in aquatic ecosystems. WF is therefore only relevant in terrestrial ecosystems. Soils absorb and retain solutes; the related benefit for human well-being is the reduction of dissolved chemicals in water resources utilised, for instance, for drinking (Dominati et al. 2010). Water filtration is an ES that is based on the percolation rate and the absorption capacity of soils controlled by many soil parameters such as clay and organic material content and composition (Drobnik et al. 2018).

The filtration effect of soils is substance-specific, and complex models are needed for a detailed assessment that considers diverse substances. When available, results for many substances can be transformed into a compound indicator representing the ES. Practically, the assessment of one relevant substance, operating as a proxy for the whole WF ES, is needed. The indicator applied in this study focusses on the filtration of nitrate as an important agricultural pollutant. Leached nitrate contaminates aquatic ecosystems leading to eutrophication and a decrease in drinking water quality. In the European Union, several policy instruments address the problem of nutrient inputs to water bodies exceeding critical values (e.g. Nitrate Directive, Water Framework Directive, and Groundwater Directive (European Union 1991, 2000, 2006b)).

The selected indicator *soil water exchange rate* in percentage per area describes the *nitrate leaching vulnerability* by the amount of water in the soil that is exchanged in 1 year. Soil depth, texture, the plant available water, the yearly evapotranspiration and precipitation, and the ground water level are important parameters for the indicator (see Müller and Waldeck (2011) for details). Hewitt et al. (2015) applied a comparable indicator to quantify the nitrate filtering for the quantification of the natural capital of soils. Jónsson et al. (2017) utilised the potential maximum amount of leached nitrate vs. the current state of nitrate leaching as a proxy to assess the current state of water filtration. Dominati et al. (2014) generated information on the ES *filtering of nutrients and contaminants* that considers nitrate and phosphate retention.

*Water flow regulation* (WFR) is a regulating ES related to the hydrological cycle and the control of floods. The human benefit is the mitigation of damages caused by floods and storms as well as drought prevention. In this study, WFR is defined as the regulating effect of ecosystems, mainly related to their abiotic components, especially soils. In contrast, CICES V5.1 explicitly defines WFR only as a biotic ES (CICES class “hydrological cycle and flood control” (CICES code 2.2.1.3)) while the abiotic part of the ES WFR can be subsumed under the CICES class “control of liquid flows” (CICES code 5.2.1.2) (Haines-Young and Potschin-Young 2018). Burkhard et al. (2014) list the ES WFR as a service regulating the water cycle and propose water storage capacity as an indicator for the potential ES supply. The water storage capacity defines the soil-related potential to regulate floods, for instance, caused by heavy rainfall. In addition, water storage capacity defines the amount of water stored in case of drought. Therefore, the indicator for the ES WFR applied in this study is *water storage capacity of the soil*, specified as the amount of potential storable soil water in millimetres. Based on the instructions by Müller and Waldeck (2011), the indicator was calculated based on key parameters of soils such as texture, bulk density, stone content, and organic matter content.

*Fresh water provision* (FWP) is a provisioning ES that has been defined in CICES C5.1 (Haines-Young and Potschin (2018) as the supply of (drinking) water from ground water sources. For the ES FWP, an assessment of the amount of available drinking water is needed (quantity). It differs from the ES WF that assesses indirect the water quality (purity, lack of harmful substances). The amount of stored ground water is not a useful indicator as it is not necessarily a function of the actual ecosystem conditions. Instead, the current contribution of the ecosystem to the water resources that can be utilised by humans is important. Therefore, the applied indicator is the mean *percolation rate* that describes the potential amount of water that is added to the ground water sources in millimetres per area. Parameters utilised in the estimation of the percolation rate are land use, precipitation and evapotranspiration (differentiated by seasons), and plant available soil water storage capacity (see Müller and Waldeck (2011) for details).

### Potential ecosystem services supply

The ES supply indicators were transformed to a relative scale with six levels, representing quantities of potential ES supply. According to Burkhard et al. (2012), the levels range from 0 to 5 indicating *no* (0), *very low* (1), *low* (2), *medium* (3), *high* (4), and *very high* (5) relevant ES supply potentials. Existing classification schemes by Müller and Waldeck (2011) were applied to define the class boundaries for the specific indicators. It must be stated that the class *no ES supply* (0) does not occur in the investigated areas (Table 3).

**Table 3:** Class boundaries used to transform the applied ecosytem service indicators to a relative scale of potential supply of the specific ecosystem services

Potential ecosystem service supply (relative scale)	0 (no)	1 (very low)	2 (low)	3 (medium)	4 (high)	5 (very high)
**Crop provision (CP)** **Indicator:** potential arable yield (*t/ha winter barley*)	0	≤ 2500	2500 to < 2875	2875 to < 3250	3250 to < 3625	≥ 3625
**Water filtration (WF)** **Indicator:** nitrate leaching vulnerability (water exchange rate) (*%/a*)	-	≥ 250	150 to < 250	100 to < 150	70 to < 100	< 70
**Water flow regulation (WFR)** Indicator: water storage capacity (*mm*)	0	< 50	50 to < 90	90 to < 140	140 to < 200	≥ 200
**Fresh water provision (FWP)** **Indicator: **percolation rate (*mm/a*)	0	< 200	200 to < 250	250 to < 300	300 to < 350	≥ 350

## Results

### Soil profile reduction

**Fig. 5:**
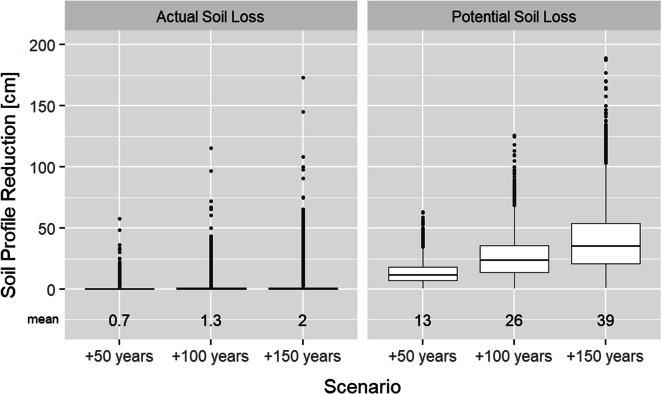
Boxplots showing the soil profile reduction for the scenarios *actual soil loss* and *potential soil loss* combined with the time steps +50, +100, and + 150 years (*cm*) included mean for all combinations (*n* = 29,181, number of raster cells in the investigation area)

Extrapolating the spatially explicit soil erosion rates into the future led to spatially explicit data on soil profile reduction. The boxplots in Fig. 5 show the soil profile reduction for the 29,181 raster cells covering the investigation area. Assuming stable loss rates, the actual soil loss rates would reduce the soil in average by 2 cm in 150 years. Based on the potential soil loss rate, the average reduction would be 13 cm after 50 years and would increase linearly to 39 cm in the +150-year scenario. The maximum annual soil loss rates would result in a profile reduction of 173 cm (actual soil loss) and 189 cm (potential soil loss) after 150 years. In all scenarios, the lowest soil loss rates resulted in profile reductions less than 1 cm.

### Ecosystem service indicators for the scenarios

The soil profile reduction resulted directly in changes in the potential ES supply. The decrease in the ES indicators *potential arable yield* and *water storage capacity* indicate a reduction of the potential ES supply (see Table 4). The increasing *nitrate leaching risk* points to a reduced potential ES supply and the increasing *percolation rate* indicates an increased potential ES supply. In contrast to the linear soil profile reduction, the indicators did not change linearly. This is due to varying soil properties within the soil profiles.

**Table 4:** Statistical parameters of the ecosystem service indicators for the initial state and the scenarios (*n* = 29,181, number of raster cells in the investigation area)

Ecosystem service indicator	Statistical parameter	Scenario						
		Initial state	Mitigated impact | actual soil loss			Structural impact | potential soil loss		
			+50 a	+100 a	+150 a	+50 a	+100 a	+150 a
Potential arable yield (*t/ha winter barley*)	Mean	3369	3328	3311	3293	2973	2590	2292
	Min	991	954	954	782	526	708	708
	Max	3845	3837	3837	3837	3788	3760	3730
	SD	465	467	474	485	557	702	807
Nitrate leaching risk (soil water exchange rate) (*%/a*)	Mean	87.7	89.7	90.7	92.8	122.4	173.4	340.2
	Min	43.6	44.1	44.1	44.1	48.3	53.3	58.3
	Max	456.1	553.8	2051.2	16250.0	13976.5	30065.1	30065.1
	SD	35.0	36.9	39.3	105.3	224.6	405.1	1086.2
Water storage capacity (*mm*)	Mean	218.4	214.5	212.9	211.2	182.8	151.1	122.9
	Min	44.0	41.8	0.0	0.0	2.2	0.0	0.0
	Max	279.4	276.6	276.6	276.6	273.8	271.1	265.5
	SD	44.6	44.4	44.8	45.5	52.0	61.4	69.4
Percolation rate (*mm/a*)	Mean	279.8	281.1	281.6	282.2	293.4	309.0	326.3
	Min	178.0	178.3	178.3	178.3	180.9	183.6	186.8
	Max	390.6	426.4	502.5	552.5	573.0	684.0	684.0
	SD	42.9	43.0	43.0	43.3	49.1	57.5	70.5

The changes in the potential ES supply in the *mitigated impact scenarios* were rather small. For instance, the average *percolation rate* increased by just 1.1 *mm/a* in 150 years (see Table 4). The largest increase in the *mitigated impact scenarios* was shown by the indicator *nitrate leaching risk*: the average *soil water exchange rate* increased from 89.7 to 92.8 *%/a* in 150 years.

In line with higher soil loss rates, the changes in the *structural impact* scenarios were much higher: Compared to the initial state, the average *water storage capacity* was reduced by 95.5 *mm* in 150 years—a decrease of 44*%*. The average *nitrate leaching risk* was nearly 4 times higher after 150 years and the *average potential arable yield* decreased significantly from 3369 to 2292 *t/ha* in 150 years. Compared to the other indicators, the changes in the *percolation rate* were the lowest in the *structural impact scenarios* (see Table 4).

### Potential supply of soil-related ecosystem services

**Fig. 6:**
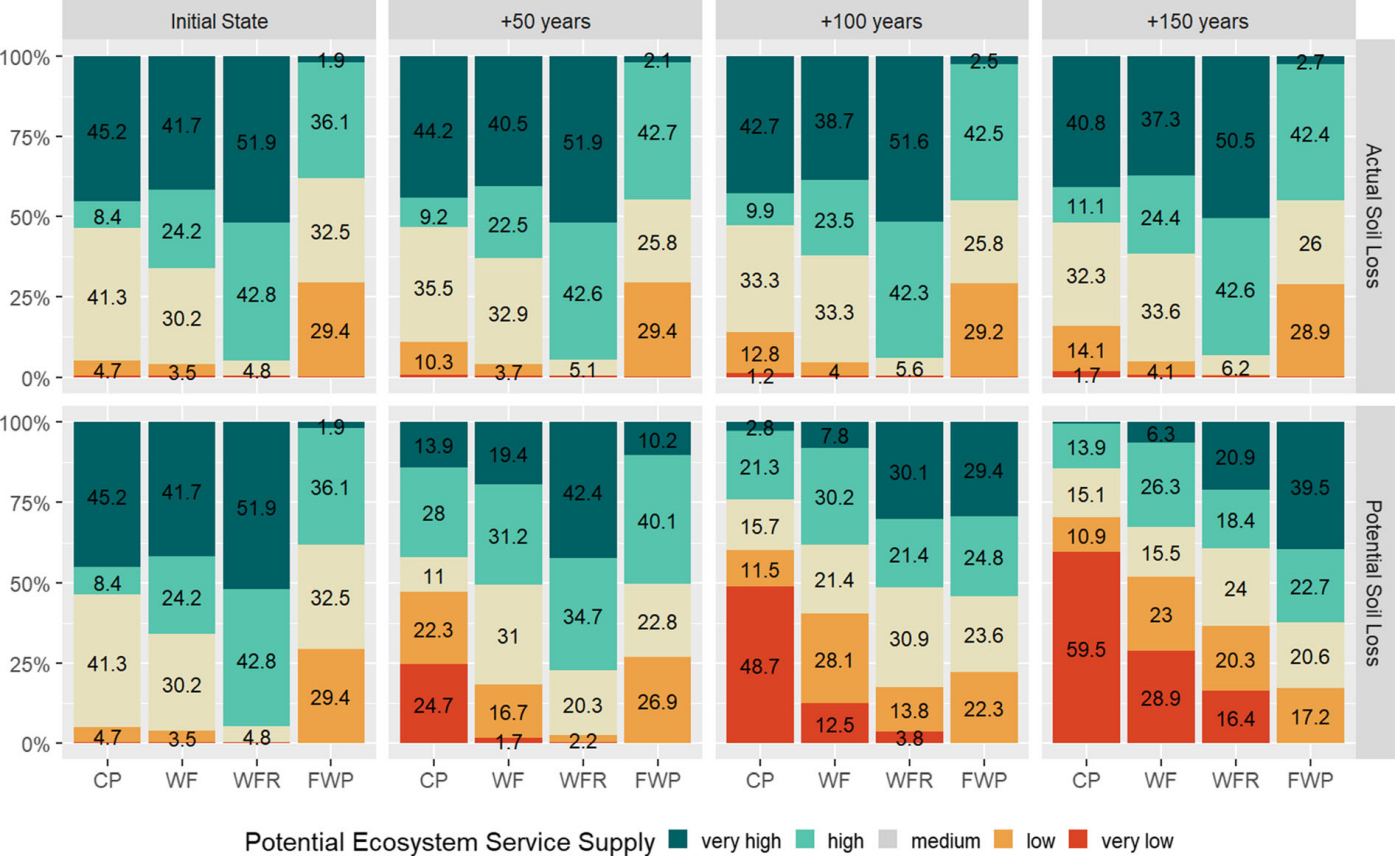
Area shares of the five specific classes of the potential ecosystem service supply for the four selected soil-related ecosystem services for the initial state and the six scenarios. CP crop provision, WF water filtration, WFR water flow regulation, FWP fresh water provision. Labels are only shown for area shares > 1% (*n* = 29,181 number of raster cells, resp. 456.5 ha)

Transforming the modelled indicators to relative scales of potential ES supply (see paragraph “Potential ecosystem services supply” in the “Material and methods” section), enables the comparison of the spatially explicit outputs of the scenarios. Figure 6 summarises this spatial information by presenting the area share of potential ES supply classes for each ES and scenario. Generally and in line with the low profile reductions, changes in the scenarios for the actual soil loss were comparably small.

In the initial state, the ES crop provision (CP) classes *very high*, *high*, and *medium* summed up to an area share of 94.7%, indicating an overall high potential ES supply. The areas categorised to these classes decreased in the scenarios, whereas the classes *low* and *very low* increased. Changes were very high in the *structural impact scenarios*: The class representing *very low* crop provision increased from an area share < 1 to 59.5% in 150 years, the class *very high* decreased to under 1%.

The changes in the ES water filtration (WF) were similar: starting with large area shares in the classes *very high* and *high* at the initial state (41.7 and 24.2%), the potential ES supply decreased intensively in the *structural impact scenarios*. The classes indicating *low* and *very low* potential ES supply for water filtration did not increase in the same magnitude as for the ES crop provision: the class *very low* increased in 150 years from an area share of 1 to 28.9% in the *structural impact scenarios*.

In the initial state, the ES water flow regulation (WFR) was the highest of all investigated soil-related ES. Due to thick soils with high water storage capacity, the area share for the classes *high* and *very high* was 42.8 and 51.9%. Compared to the ES crop provision and water filtration, the decrease of areas assigned to the classes *high* and *very high* was less: after 150 years still 20.9% of the investigation area showed a *very high* potential to supply this service.

In contrast to the ES crop provision, water filtration, and water flow regulation, the potential supply for the ES fresh water provision (FWP) increased with declining soil profile depths. In the *mitigated impact scenarios*, the changes in 150 years were marginal. In the *structural impact scenarios*, the area share of the two upper classes increased from combined 38 to 62.2%. It must be noted that in no scenario not a single grid cell falls into the class *very low.*

**Fig. 7:**
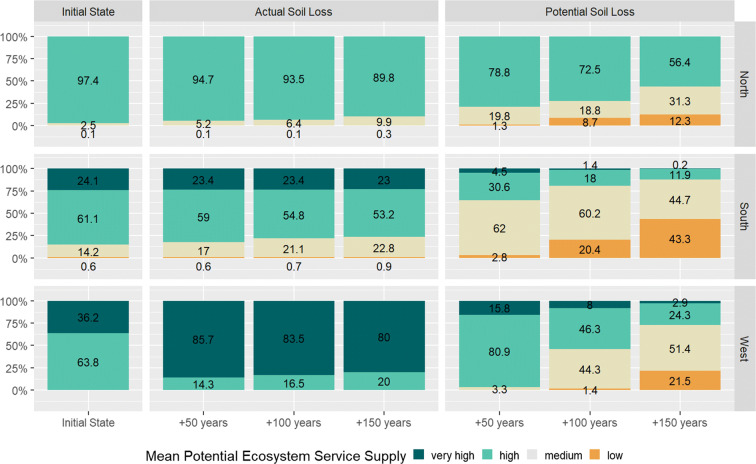
Area shares of the classes of the mean potential ecosystem service supply for the initial state and the six scenarios in the three investigation regions. (*n* = 29,181 number of raster cells, 465.5 *ha*; northern region: 8 811 cells (137.7 *ha*); western region: 1811 cells (28.4 *ha*), southern region: 18 559 cells (298.3 *ha*))

Figure 7 summarises the changes in the potential ES supply by showing the average potential ES supply of all investigated soil-related ES for the regions North, West, and South. Based on the specific classification for the investigated ES, the averages generally increased the area shares of the classes *high* and *medium.* In all scenarios, the average of the potential ES supply eliminated the occurrence of the class *very low*.

The initial average in the potential ES supply differs between the regions. In the small western region (28.4 *ha*), only the potential ES supply classes *high* and *very high* occurred. In the northern region, nearly the whole area of 137.7 *ha* cropland had a *high* potential ES supply. The southern region showed a more diverse pattern: 14.2% of the 298.3 *ha* of investigated cropland fell into the class of *medium* ES supply and 24.1% in the class *very high* while the largest area share (61.6%) was assessed to have a *high* ES supply.

In the *mitigated impact scenarios*, the changes in the average potential ES supply with time were minor. In the northern region, the area share of the class *medium* increased from 5.2 to 9.9%, and in the southern region from 17 to 22.8%. In the western region, only the classes *high* and *very high* occurred in the *mitigated impact scenarios* and a bigger share fell into the highest class of potential ES supply than in the initial state. This surprising result can be traced back to the specific soil composition and properties in this region in combination with low loss rates in the *mitigated impact scenarios*. This resulted in an increase in the ES fresh water supply while all other ES stayed stable.

Considering the *structural impact scenarios*, the distribution shifted in all regions from large shares of the classes *high* and *medium* to the classes *medium* and *low*. The decrease was biggest in the southern region: in the scenario for +150 years, the proportion of cropland with low ES supply was 43.3%, while 11.9% were still in the class representing a *high* potential supply of soil-related ES. In the northern region, the changes were comparable and showed a shift from large proportions of areas with *high* potential ES supply to areas with *medium* and *low* potential supply, while still more than the half of the area (56.4%) was assigned to the class *high.*

The largest differences between the scenarios for mitigated and structural impact were observed in the western region. Projecting the actual soil loss rates into the future led to an increase in the potential service supply in comparison to the initial state: after 150 years, 80% of the investigated cropland showed still a *very high* service supply. Assuming potential soil loss rates, the potential ES supply decreased: after 100 years, 44.3% of the cropland was assigned to the class *medium* potential ES supply, after 150 years, the class *medium* increased to an area share of 51.4% and 21.5% of the cropland in the western region fell into the class *low* potential ES supply.

## Discussion

### Why a new assessment approach?

Soils are fundamental parts of terrestrial ecosystems, relevant for the supply of various ES and threatened by degradation processes, which are often driven by human activities. Therefore, the protection of soil resources is mandatory to secure ES supply. Appropriate policies, sustainable management concepts, and scientific frameworks for the assessment of soil resources and their services are needed to achieve this objective. As stated by Robinson et al. (2017), two fundamentally different concepts can be applied to assess soils: (a) the assessment of soil as an “individual asset” and (b) the recognition of soil as a relevant part of ecosystems in an ES approach.

Existing approaches treating soil as an asset focus on the natural capital of soils and address the stock changes. Robinson et al. (2009) define soil natural capital as a quantifiable stock of organised biotic and abiotic mass containing energy. A related framework published by Robinson et al. (2017) proposes mass balances between soil erosion and formation as a key element in the assessment of soil natural capital and presents a mass account for soil stocks in the EU which is also applicable for monitoring purposes.

The importance of soils in the ES concept is widely discussed (Daily et al. 1997; Palm et al. 2007; Robinson et al. 2009; Dominati et al. 2010; Robinson and Lebron 2010; Robinson et al. 2012). Related frameworks propose the assessment of ES provided by soils (Robinson et al. 2013; Robinson et al. 2014; Hewitt et al. 2015; Jónsson et al. 2017). Robinson et al. (2014) state that soils are mostly underrated (“poorly addressed”) in ES approaches. In addition, ES-centred approaches generally do not address changes in the service supply due to degradation of soils.

Within the ES concept, soil erosion is mainly addressed by the regulating ES *control of erosion rate* (often named *erosion regulation* or also known as *soil retention*). The service supply is defined as the mitigation of a structural impact and a function of ground cover by plants and soil management (Guerra et al. 2014). Many general studies and reviews on ES emphasise the impact of soil erosion on soils and the ES they provide (e.g. Daily et al. 1997; Reid 2005; Hu et al. 2019). Nevertheless, no framework explicitly addresses the changes in soil-related ES by soil erosion nor the mitigated impacts on these services by the ES *control of erosion rates*. Appropriately, one objective of this study was the development of an assessment approach addressing this problem and its application. The new approach presented here enables (a) the comparison of changes in the supply of soil-related ES and their modification through soil erosion in time and (b) the direct comparison of scenarios representing varying future degradation states of soils.

### Long-term impacts on soil-related ecosystem services by soil erosion at the landscape scale

The study was conducted at the landscape scale, while the resolution of the spatially explicit modelling and assessment of the soil-related ES was very detailed (12.5 m raster cell size). The specific investigation areas in Northern Germany all represent landscapes typically prone to soil erosion by water, while they differ in topography, soils, and management practices. Due to their explicit vulnerability to soil erosion by water, only cropland was included in this study. Nevertheless, landscape is the appropriate scale to address soil erosion as its occurrence is related to landscape patterns (Ouyang et al. 2010). Besides this, landscape is a relevant and suitable scale for the aggregation of ES assessments. At the same time, results aggregated at the landscape scale should not be generated as a simple average of the spatially explicit results produced on a finer scale. Information on potential ES supply at a landscape scale must reflect its characteristics. Therefore, maps presenting the spatial distribution of different ES supply classes and figures summarising the area shares of ES supply classes—and in this study the change in ES supply—are powerful tools for the transportation of aggregated results.

The results show that the impact of future potential soil erosion on the potential supply of the selected soil-related ES is generally high. At the same time, losses in ES supply differ between the investigation areas representing different landscapes. The aggregated results for the specific investigation areas can be linked to the loss rates, management practices, and soil conditions.

The soils eroded heavily in the scenarios for the potential soil loss, resulting in relevant decreases in potential ES supply in three out of four services. At the same time, the regulating ES fresh water provision (FWP) increased. The values for the average potential ES supply generally suppressed the increase of the ES FWP in the northern and southern region. Considering the actual soil loss, the long-term changes in the potential ES supply were generally low for all four selected soil-related ES, while the ES crop provision reacted most sensitively. At the same time, the actual soil loss rates in the western region led to an increase in the average ES supply due to an increase in the ES FWP, while the other soil-related ES stayed stable.

Combining the results of the scenarios with different levels of impact emphasised the relevance of the ES control of erosion rates: mitigating the structural impact prevents not only the loss of soil material but also the loss of soil-related ES. The presented assessment approach enables the quantification of the mitigation effect. In the case study presented here, after 150 years, the area with a *very high* average ES supply will be more than 65 times smaller in case the ES control of erosion rates is neglected. Especially in the southern region, characterised by high potential loss rates, medium actual loss rates, and at least partly shallow soils, the mitigated loss in areas with *very high* and *high* potential ES supply was immense.

### Contrary response of ecosystem services to changes in soil properties

The case study results demonstrate the importance of including various ES in integrated ES assessments as they respond differently to changes in soil properties. In our case study, the indicator applied for the ES FWP *percolation rate* represent the water quantity potentially added to a ground water source. While the potential surplus in water quantity increased with time in the scenarios, the ES water filtration indicated degradation in the water quality. Both ES are strongly interrelated portraying different aspects of water resources. However, the ES for water quantity and quality showed different trends. This kind of negative correlation between ES should be addressed in ES analyses. This outcome emphasises that the often demanded integration of many ES must be accompanied by a detailed analysis of thematic interlinkages between the assessed ES (Gissi et al. 2018), while the double-counting of individual ES must be avoided (Burkhard et al. 2014).

### Uncertainties and assumptions

This study combines a variety of datasets with varying uncertainties that must be taken into account in the assessment of these results. The utilised soil map of Lower Saxony in the scale of 1:50,000 is the currently best available geodata on soils with complete soil profile descriptions for the investigation area. Nevertheless, the map scale indicates uncertainties: While in reality soil is a phenomenon constantly changing in space, the map provides generalised soil profile descriptions related to specific map units that were transformed to a raster dataset with a resolution of 12.5 *m* in this study. Minor changes in soil composition at small scale are neglected, but datasets with a higher resolution does not exist.

All methods applied for the calculation of the ES indicators are estimations based on soil and climate data (Müller and Waldeck 2011). The same methods and data are used by soil conservation authorities in Lower Saxony. Therefore, it can be confirmed that the data used in this study is suitable for the methods and the results are useful and reliable.

The spatially explicit datasets on loss rates are additional sources of uncertainty: Steinhoff-Knopp and Burkhard (2018b) estimate that the error rate for the actual loss rates obtained in the Lower Saxonian soil erosion monitoring programme is 15%. The utilised data on potential soil loss rates are spatially explicit and were calculated by utilising the USLE as the German standard (Deutsches Institut für Normung e.V. 2017). The USLE and its multiple adaptations are the most often used soil erosion models delivering reliable information on soil loss. Nevertheless, Steinhoff-Knopp and Burkhard (2018a) stated that the estimated loss rates are too high for Northern Germany, which leads to the overestimation of the decrease in soil-related ES in this study.

For the development and testing of the general suitability of the assessment approach in this case study, some assumptions were made. First of all, constant soil loss rates were extrapolated into the future to create scenarios of degraded soils. Considering climate change and changes in agricultural practice, it is very unlikely that the potential and actual soil loss rates will be stable over the next 150 years. In addition, the utilised soil profile reduction approach does not reflect soil processes as soil formation or the reorganisation of soil structures after topsoil erosion. Also, changes in the soils and their services due to the sedimentation of eroded soil particles (colluvium forming) or due to agricultural practices (e.g. ploughing) were not addressed in this case study.

The focus of this study was to test the general suitability of a new approach. Therefore, the application of feasible and reliable methods based on robust data was preferred. All datasets utilised in this study provide reliable information and the final assessment of the classified potential ES supply provides valid and useful results with assessable and acceptable uncertainties. In total, the model chain leads to reliable results. Due to high potential soil loss rates and simplifications in the soil profile reduction, the amount of the estimated changes in soil-related ES in the scenarios must be considered partly hypothetical. Nevertheless, the general results and trends are useful and valid and underpin the capabilities of the assessment approach developed in this study.

Some of the uncertainties and assumptions can be addressed in future studies. More detailed soil data can be generated in time-consuming soil surveys. Taking into account a soil formation rate as done by Verheijen et al. (2009), the integration of spatially explicit data on the accumulation of detached soil or even the application of a coupled landscape and soil evolution model (e.g. SaLEM, Bock et al. 2018) will enable a dynamic modelling and mapping of erosion-related changes in soil structure and properties. A combination with more detailed studies on ecosystem condition and further ecosystem services, assessing their trade-offs and synergies, would further enhance the application potential of this study.

## Conclusions

Taking up the research questions from the “Introduction” section, it can be stated that the assessment approach developed and tested in this study is a suitable tool for analysing the long-term effect of the ES control of erosion rates on soil-related ES. The potential ES supply differs significantly between the scenarios with different levels of impact: In the *structural impact* scenarios, the high potential soil loss rates lead to large decreases in the potential ES supply in three out of four considered soil-related ES. Therefore, the mean potential ES supply decreases significantly. In the *mitigated impact* scenarios, the actual loss rates are very low. Accordingly, the changes in the mean potential ES supply are generally low. The overall mitigated loss in potential ES supply by the ES control of erosion rates is large. It is the difference between the potential ES supply in the scenarios with different levels of impacts.

The decrease in potential ES supply is largest for the ES crop provision, as the applied indicator reacts very sensitively to soil degradation. The decreases in the ES water filtration and fresh water provision are smaller whereas the ES fresh water provision increases due to increasing percolation rates.

Generally, the results in the investigation areas are similar: large decreases in potential ES supply in the *structural impact* scenarios and small decreases in the *mitigated impact* scenarios. Only the western region reacts differently: In the *mitigated impact* scenarios, the mean potential ES supply increases due to the increased supply of the ES *fresh water provision.* This effect can be traced back to the specific combination of soil composition and loss rates.

The results on changes in the potential ES supply in the scenarios with different levels of impact emphasise the importance of sustainable farming and soil management for the preservation of soil functions and related ES. Furthermore, using scenarios that represent the impact of land management practices of varying intensities, the approach can be a powerful tool to assess the sustainability of land use practices. The approach can also be adapted to any soil threat, such as soil compaction or soil sealing. Respective assessments should be based on suitable and detailed data. Indicators for relevant soil-related ES as well as a set of realistic scenarios with appropriate descriptions of the processes degrading the soils should be selected. The scenario approach can be transferred to any ecosystem component and associated threats.
